# Predisposing Factors of Tuberculosis Infection in Systemic Lupus Erythematosus Patients: A Single-Center Case-Control Study

**DOI:** 10.7759/cureus.26410

**Published:** 2022-06-28

**Authors:** Ivan Damara, Anna Ariane, Kevin Winston

**Affiliations:** 1 Internal Medicine, Cipto Mangunkusumo National General Hospital, Universitas Indonesia, Jakarta, IDN; 2 Rheumatology, Cipto Mangunkusumo National General Hospital, Universitas Indonesia, Jakarta, IDN

**Keywords:** systemic lupus erythematosus, tuberculosis, sledai, corticosteroid, lupus nephritis

## Abstract

Introduction: Indonesia has the second-highest tuberculosis cases in the world, according to the global WHO tuberculosis report, amounting to approximately 10% of the world's tuberculosis cases. Systemic lupus erythematosus (SLE) patients are at an increased risk for tuberculosis infection. This research aims to analyze the association between corticosteroid pulse dose, corticosteroid cumulative dose, SLE disease duration, Systemic Lupus Erythematosus Disease Activity Index (SLEDAI) score, and lupus nephritis status with the development of tuberculosis in SLE patients.

Methods: This research was a matched case-control study to identify risk factors of tuberculosis infection in SLE patients. Data were taken from medical records of Cipto Mangunkusumo National General Hospital, a national tertiary hospital. Inclusion criteria were patients who meet the Systemic Lupus Erythematosus International Collaborating Clinics (SLICC) 2012 criteria of SLE in the period of 2012-2016 or patients who meet the SLICC 2012 SLE criteria and developed tuberculosis between 2012 and 2016. Statistical analyses used were bivariate analysis and correlation analysis. All statistical analyses were conducted using SPSS software (IBM Corp., Armonk, NY). All statistical analyses were defined as statistically significant when the p-value was less than 0.05.

Results: A total of 48 SLE patients were included from medical records consisting of 24 SLE patients with tuberculosis infection and controls of 24 SLE patients without tuberculosis infection. In this study, it was observed that the presence of lupus nephritis (p = 0.001), administration of pulse corticosteroids (p = 0.048), high corticosteroid cumulative dose (p = 0.001), and high SLEDAI score (p = 0.003) were associated with tuberculosis infection. Correlation analysis showed that all of these variables had a weak positive correlation with tuberculosis infection in SLE patients.

Conclusion: SLE patients with lupus nephritis, administration of pulse corticosteroids, high cumulative corticosteroid dose, and high SLEDAI score have a higher risk of tuberculosis infection. Clinicians and patients should be aware of these risk factors in SLE patients to prevent tuberculosis infection. Corticosteroid pulse dose should be avoided in SLE patients and if it is needed, tuberculosis prophylaxis may be considered.

## Introduction

According to the WHO, the global death rate of tuberculosis in the year 2020 was around 1.3 million [1]. Currently, Indonesia represents the third-highest tuberculosis burden country after India and China [1]. Many studies have shown that tuberculosis infection has high mortality and morbidity [2-4].

While immunocompromised patients, such as systemic lupus erythematosus (SLE) patients, are at a risk for common infections, SLE patients in Indonesia are also at risk for tuberculosis infection due to the high prevalence of tuberculosis [5]. Thus, this is a major issue for SLE patients in Indonesia. Further aggravating this issue is that many SLE patients use corticosteroids, which may also contribute to tuberculosis infection [6,7].

Currently, there is a paucity of data regarding tuberculosis infection in SLE patients in Indonesia despite the high prevalence of tuberculosis infection. Thus, there is an urgent need for data regarding this issue to increase patient care and awareness of clinicians since diagnosis of tuberculosis is often delayed in SLE patients. This research aims to analyze the association between corticosteroid pulse dose, corticosteroid cumulative dose, SLE disease duration, Systemic Lupus Erythematosus Disease Activity Index (SLEDAI) score, and lupus nephritis status with the development of tuberculosis in SLE patients.

## Materials and methods

This research was a matched case-control study to identify risk factors of tuberculosis infection in SLE patients. Data were taken from medical records of Cipto Mangunkusumo National General Hospital, a national tertiary hospital. The population of this research was SLE patients from February 2012 to February 2016.

Inclusion criteria were patients who met the Systemic Lupus Erythematosus International Collaborating Clinics (SLICC) 2012 criteria of SLE from 2012 to 2016 or patients who met the SLICC 2012 criteria of SLE and developed tuberculosis between 2012 and 2016 [8]. The exclusion criteria were patients who were lost of follow-up and patients with comorbidities (HIV, chronic kidney disease, and diabetes).

Independent variables analyzed in this study were corticosteroid pulse dose, cumulative dose of corticosteroid, SLEDAI score, SLE disease duration, and lupus nephritis status. Corticosteroid pulse dose was defined as the administration of intravenous corticosteroid within the last three months. The cumulative dose of corticosteroid was defined as the total dose of corticosteroid taken by the patient in the period of three months. SLE disease duration was defined as the duration of SLE disease since SLE diagnosis. SLEDAI score is divided into no activity (SLEDAI: 0), mild activity (SLEDAI: 1-5), moderate activity (SLEDAI: 6-10), high activity (SLEDAI: 11-19), and very high activity (SLEDAI ≥ 20) [9]. The diagnosis of tuberculosis infection in this study was made by using acid-fast bacilli test, Mantoux test, GeneXpert test, magnetic resonance imaging (MRI), CT scan, spinal tap, biopsy, X-ray with clinical diagnosis, and polymerase chain reaction (PCR).

Data from medical records were entered and processed using SPSS software for Windows version 20.0 (IBM Corp., Armonk, NY). Bivariate analysis was conducted to analyze the association between independent and dependent factors. Bivariate analysis for categorical data utilized the chi-square test when a minimum of 20% of cells possess the expected count of less than five. If this condition was not fulfilled, Fisher’s exact test was used instead for the 2 x 2 table. Meanwhile, bivariate analysis for numeric-categorical data used the Mann-Whitney U test if the data were not normally distributed and the independent t-test when the data were normally distributed. The normal distribution of the data was assessed using the Kolmogorov-Smirnov test or Shapiro-Wilk test. For correlation analysis, Pearson’s correlation test was used for normally distributed data. Meanwhile, the Spearman correlation test was used for data without normal distribution. All statistical analyses were defined as statistically significant when the p-value was less than 0.05.

## Results

A total of 48 patients were included from medical records consisting of 24 SLE patients who developed tuberculosis infection and controls of 24 SLE patients without tuberculosis infection development (Table 1). Matching was performed for age and sex.

**Table 1:** Subject characteristics of SLE patients who developed tuberculosis infection and SLE patients who did not develop tuberculosis infection (n = 48) Categorical data are presented as n (%) and continuous data as mean ± SD for data with normal distribution or as median (min-max) for data with non-normal distribution. SLE: systemic lupus erythematosus.

Variables	SLE with tuberculosis infection (n = 24)	SLE without tuberculosis infection (n = 24)
Age (years)	26 (12-41)	32 (16-46)
Female (n)	23 (95.83%)	23 (95.83%)
Musculoskeletal SLE involvement (n)	22 (91.66%)	20 (83.33%)
Mucocutaneous SLE involvement (n)	4 (16.66%)	11 (45.83%)
Hematology SLE involvement (n)	7 (29.16%)	4 (16.66%)
Kidney SLE involvement (n)	13 (54.16%)	2 (8.3%)
Neuropsychiatry SLE involvement (n)	3 (12.5%)	1 (4.16%)
Vasculitis SLE involvement (n)	1 (4.16%)	0 (0%)
Serositis SLE involvement (n)	1 (4.16%)	0 (0%)
Hemoglobin (g/dL)	9.683 ± 2.69	10.929 ± 2.151
Leukocytes (/mL)	6,495 (3,020-12,670)	6,895 (2,230-17,240)
Thrombocytes (/µL)	302,500 (27,500-651,000)	333,000 (10,000-302,500)
Erythrocyte sedimentation rate (mm/hour)	80 (18-148)	36 (6-120)
Pulse dose (n)	7 (29.16%)	1 (4.16%)
Cumulative corticosteroid dose (mg)	2,115 (747-3,483)	990 (222-1758)
Hydroxychloroquine (n)	4 (16.66%)	7 (29.16%)
Azathioprine (n)	4 (16.66%)	6 (25%)
Mycophenolate mofetil (n)	6 (25%)	2 (8.33%)
Lupus nephritis (n)	13 (54.16%)	2 (8.3%)

The median age of SLE patients who developed tuberculosis infection was 26 years ranging from 12 to 41 years. Meanwhile, the median age of the control group was 32 years ranging from 16 to 46 years. The majority of the patients in both groups had musculoskeletal SLE involvement. Mean hemoglobin appeared to be lower in SLE patients who developed tuberculosis infection than in controls (9.683 g/dL vs. 10.929 g/dL). In contrast, the control group had a lower cumulative corticosteroid dose. Among the 24 patients with SLE and tuberculosis infection, seven patients (around 25%) received a pulse dose of corticosteroid with varying doses (one patient received 50 mg, three patients received 65 mg, two patients received 125 mg, and one patient received 250 mg). In contrast, among 24 controls, only one patient had been administered with pulse dose of corticosteroid.

A total of seven patients showed positive tuberculosis infection results using the acid-fast stain test, three patients with the Mantoux test, four patients with the GeneXpert test, and two patients were diagnosed with PCR. The additional diagnosis modalities used for the remaining eight patients with extrapulmonary tuberculosis were as follows: MRI and spinal tap for meningoencephalitis tuberculosis, CT scan and spinal tap for spondylitis tuberculosis, biopsy for lymphadenitis tuberculosis, and X-ray along with clinical diagnosis for miliary tuberculosis. Of 24 patients, 16 patients (67%) suffered from pulmonary tuberculosis and eight patients (33%) experienced extrapulmonary tuberculosis (Table 2).

**Table 2:** Sites of tuberculosis infections in SLE patients SLE: systemic lupus erythematosus.

Number of patients (%)	Sites of tuberculosis infections
3 (12.50%)	Miliary
2 (8.33%)	Lymphadenitis
1 (4.16%)	Meningoencephalitis
1 (4.16%)	Spondylitis
1 (4.16%)	Myelitis
16 (66.67%)	Pulmonary

Based on the SLEDAI score, 14 patients had mild lupus activity, 15 patients had moderate lupus activity, nine patients had high lupus activity, and 10 patients had very high lupus activity (Table 3).

**Table 3:** SLEDAI score classifications of SLE patients (n = 48) SLE: systemic lupus erythematosus; SLEDAI: Systemic Lupus Erythematosus Disease Activity Index.

SLEDAI score classification	N (%)
Mild SLE activity	14 (29.17%)
Moderate SLE activity	15 (31.25%)
High SLE activity	9 (18.75%)
Very high SLE activity	10 (20.83%)

The cumulative dose of corticosteroids of an average of 2,115 mg within three months showed a positive association with the development of tuberculosis infection (p < 0.001) using the Mann-Whitney U test (Table 4). SLE patients without tuberculosis infection were observed to have only 990 mg of cumulative dose. This finding indicated that a cumulative dose of corticosteroid was a risk factor for tuberculosis infection development in SLE patients. The presence of lupus nephritis was significantly higher compared to controls (p < 0.001). SLEDAI score was also a factor associated with tuberculosis infection (p = 0.003). However, SLE disease duration and corticosteroid pulse dose were not associated with tuberculosis infection based on the analysis.

**Table 4:** Comparison of variables of SLE patients with tuberculosis infection versus SLE patients without tuberculosis infection Data are presented in average and analyzed by (a) chi-squared test, (b) Mann-Whitney U test, and (c) independent t-test. SLE: systemic lupus erythematosus; SLEDAI: Systemic Lupus Erythematosus Disease Activity Index.

Variables	SLE patients with tuberculosis	SLE patients without tuberculosis	P-value
Pulse dose (n)	7	1	0.048^a^
Lupus nephritis (n)	13	2	0.001^a^
Cumulative dose of corticosteroid (mg)	2,115 (747-3,483)	990 (222-1,758)	0.001^b^
SLE disease duration (years)	2 (0-14)	3 (0-24)	0.192^b^
SLEDAI score	15.5 (2-38)	6 (3-25)	0.003^b^
Hemoglobin (g/dL)	9.683 ± 2.69	10.929 ± 2.151	0.083^c^

In the correlation analysis, it was found that SLEDAI score (correlation coefficient: 0.437), corticosteroid dose (correlation coefficient: 0.434), and lupus nephritis status (correlation coefficient: 0.494) had correlations with tuberculosis infection development in SLE patients (Table 5). Based on the correlation coefficient strengths, these three variables had moderate correlation strength. Meanwhile, age, hemoglobin level, and SLE lupus duration were not correlated with tuberculosis infection development in SLE patients.

**Table 5:** Correlation between variables and tuberculosis infection development in SLE patients SLEDAI: Systemic Lupus Erythematosus Disease Activity Index.

Variables	Correlation coefficients	P-value
Age	-0.267	0.067
Lupus duration	-0.190	0.196
Hemoglobin	-0.244	0.095
SLEDAI score	0.437	0.002
Cumulative dose of corticosteroid	0.434	0.002
Lupus nephritis status	0.494	<0.001

## Discussion

From the matched case-control in this study, we observed that SLE patients who developed tuberculosis infection had a higher cumulative dose of corticosteroid and SLEDAI score when compared with controls. There was also a higher proportion of patients who received corticosteroid pulse dose and had lupus nephritis status in the group that developed tuberculosis than in the control group. All of these indicate that a high cumulative dose of corticosteroids, high SLEDAI score, receiving corticosteroid pulse dose, and having lupus nephritis are risk factors for tuberculosis infection in SLE patients. The correlation analysis showed that there were weak correlations between SLEDAI score, cumulative corticosteroid dose, corticosteroid pulse dose, and lupus nephritis status with tuberculosis infection developments.

Prolonged corticosteroid use, especially with high doses, has been shown in many studies to be a risk for both serious and opportunistic infections [10,11]. Our study shows that corticosteroids that have a beneficial effect on SLE patients in reducing SLE disease activity may cause tuberculosis infection. Many studies have found corticosteroids to be a significant risk factor for tuberculosis infection in SLE patients [12-14]. Tam et al. [13] demonstrated in their research that a high cumulative dose for one year is an independent risk factor for tuberculosis with an increase of 22% increment risk for every gram of corticosteroid. Meanwhile, Yun et al. showed that the mean daily dose of SLE complicated with tuberculosis is significantly higher than that without infection [15]. This study found similar results in the effect of cumulative steroid dose on tuberculosis infection.

Several studies conducted in Hong Kong, Singapore, and Korea, which also have a high incidence of tuberculosis, have similarly concluded that the corticosteroid pulse dose and cumulative dose of corticosteroid are significant risk factors for tuberculosis infection in SLE patients [13,14]. Hong Kong is also an endemic country of tuberculosis and thus could possibly represent other endemic countries such as Indonesia [1]. Around one-third of the patients who suffer from SLE and tuberculosis in Hong Kong were also receiving other immunosuppressive therapies. Among these patients, nine patients were on azathioprine, three patients were on oral cyclophosphamide, and five patients were on cyclosporine. However, the study concluded no correlation between these drugs with the development of tuberculosis [13].

This study showed a significant correlation between corticosteroid pulse dose with tuberculosis infection development in SLE patients and is in line with a previous study done by Tam et al. [13]. In our study, tuberculosis infection developed no longer than three months after the administration of corticosteroid pulse dose. This finding suggests that pulse dose is a significant risk factor for tuberculosis infection in SLE patients in endemic countries in the first few weeks. In turn, this might lead to the suggestion of isoniazid prophylaxis after pulse dose steroid. Isoniazid prophylaxis has been found to reduce the risk of tuberculosis infection in SLE by 70-82% in previous studies [16,17]. Based on this study, tuberculosis prophylaxis in SLE patients may be considered in those who receive pulse doses, particularly in endemic countries.

Many studies have found lupus nephritis to be a risk factor and predictor of major infection in SLE patients [18,19]. In one study done in north India, which is also a high burden country for tuberculosis, it is shown that among other infections, tuberculosis is the most common infection with around 22% of total cases [19]. A study in Hong Kong, which is also an endemic country for tuberculosis, shows a 2.26 times risk of tuberculosis infection in SLE when lupus nephritis is present [13]. It is also important to note that lupus nephritis has been found to increase infection risk not only for tuberculosis but also for other pathogens [20].

In this study, lupus nephritis is also observed to be a risk factor for tuberculosis infection in SLE patients. The reason why lupus nephritis is associated with tuberculosis infection is not currently clear. It is speculated that as lupus nephritis patients are aggressively treated with immunosuppressants, these further increase the risk of infections.

Finally, we found that a high SLEDAI score is associated with tuberculosis infection. A higher SLEDAI score means higher SLE disease activity. There may be worse immune dysregulation in SLE patients with high SLEDAI scores, resulting in higher tuberculosis infection. An observational study by Ahmmed et al. with 230 patients showed that a SLEDAI score > 12 was a risk factor for tuberculosis infection [21]. A summary of findings from other studies is presented in Table 6.

**Table 6:** Summary of findings from other studies on SLE patients SLE: systemic lupus erythematosus; SLEDAI: Systemic Lupus Erythematosus Disease Activity Index.

Authors	Year	Country	Study design	Outcomes measured	Factors associated with outcomes
Tam et al. [13]	2002	Hong Kong	Case-control	Tuberculosis Infection	Organic brain syndrome, vasculitis nephritis, receiving pulse methylprednisolone, and receiving a higher cumulative dose of prednisolone
Ruiz-Irastorza et al. [12]	2009	Spain	Nested case-control	Serious infections (*Escherichia coli,* *Staphylococcus aureus*, *Mycobacterium tuberculosis*, and *Streptococcus pneumoniae*)	Treatment with antimalarials, prednisone dose, SLE lung involvement
Feldman et al. [18]	2015	USA	Retrospective cohort	Serious infections (bacteremia, cellulitis, pneumonia, pyelonephritis, osteomyelitis, septic arthritis, endocarditis, aspergillosis, cryptococcosis, histoplasmosis, pneumocystosis, herpes zoster, cytomegalovirus, varicella-zoster, influenza, tuberculosis, and non-tuberculous mycobacteria)	Males, blacks compared to whites, glucocorticoid users, immunosuppressive users
Ahmmed et al. [21]	2019	Bangladesh	Unknown	Tuberculosis infection	High disease activity disease (SLEDAI score > 12), total intake of prednisolone > 1,000mg
González-Naranjo et al. [22]	2021	Colombia	Case-control	Tuberculosis infection	Lymphopenia for 12 months, cumulative glucocorticoid dose ≥ 1,830 mg, and been treated with ≥ 2 immunosuppressants during the last 12 months

Based on the results of this study, SLE patients are recommended to take precautionary measures to prevent tuberculosis infection. Primary care providers and rheumatologists need to also increase awareness of SLE patients regarding potential tuberculosis infection from corticosteroid use since many SLE patients require lifelong corticosteroid use. If possible, the corticosteroid pulse dose should be avoided. If SLE patients require a corticosteroid pulse dose, tuberculosis prophylaxis may be considered. All in all, the authors hypothesize that heightened awareness, prompt diagnosis, and early treatment of tuberculosis infection are more important and clinically feasible generally than long-term tuberculosis prophylaxis in SLE patients due to polypharmacy in SLE patients.

Study limitations

Being a case-control and a single-center study, we could not eliminate all biases in this study. Also, as this is a case-control study, no causality could be determined between the risk factors and tuberculosis infection development. Finally, three of the SLE patients who developed tuberculosis were diagnosed with the Mantoux test, and two patients were diagnosed with PCR, which may show false-positive results.

## Conclusions

SLE patients with lupus nephritis, administration of pulse corticosteroids, high cumulative corticosteroid dose, and high SLEDAI score have a higher risk of tuberculosis infection. Clinicians and patients should be aware of these risk factors to prevent tuberculosis infection. Corticosteroid pulse dose should be avoided in SLE patients and if it is needed, tuberculosis prophylaxis may be considered.
